# A Rare Complication of Tuberculous Meningitis Pediatric Anterior Glenohumeral Instability

**DOI:** 10.1155/2012/385782

**Published:** 2012-12-17

**Authors:** Kerem Bilsel, Mehmet Erdil, Mehmet Elmadag, Hasan H. Ceylan, Derya Celik, Ibrahim Tuncay

**Affiliations:** ^1^Department of Orthopaedics and Traumatology, Medical Faculty Hospital, Bezmialem Vakif University, Fatih, 34093 Istanbul, Turkey; ^2^Division of Physiotherapy and Rehabilitation, Faculty of Health Sciences, Istanbul University, 34740 Bakırköy, Istanbul, Turkey

## Abstract

Dislocation and instability of the shoulder joint are rare occurrences in childhood. Traumatic, infectious, congenital, and neuromuscular causes of pediatric recurrent shoulder dislocations are reported before. Central nervous system infection in infancy may be a reason for shoulder instability during childhood. This situation, which causes a disability for children, can be treated successfully with arthroscopic stabilization of the shoulder and postoperative effective rehabilitation protocols. Tuberculous meningitis may be a reason for neuromuscular shoulder instability. We describe a 12-year-old child with a recurrent anterior instability of the shoulder, which developed after tuberculous meningitis at 18 months of age. We applied arthroscopic treatment and stabilized the joint.

## 1. Introduction

Glenohumeral instability is an inability to maintain the humeral head centered in the glenoid fossa. Clinical cases of instability can be classified according to the degree of instability, the direction of instability, and the circumstances under which they occur like congenital, neuromuscular, voluntary, traumatic, and atraumatic recurrent instability [1].

Dislocation and instability of the shoulder joint are rare occurrences in children. Dislocations of the shoulder in infants have been reported previously and were either congenital dislocation with associated anomalies of the glenohumeral joint or with dislocations from Erb's palsy or septic arthritis [2].

Neuromuscular causes of shoulder instability have been reported as a recurrent dislocation developed after encephalitis, cerebral palsy, and brachial plexus birth injuries [3–6]. Anterior subglenoid dislocation of the shoulder in infant following pneumococcal meningitis has also been presented as a case report of a 7-month-old boy [7]. In this report, we discuss a 12-year-old child with a recurrent anterior instability of the shoulder, which developed after tuberculous meningitis at 18 months of age. This is the first case report of a child with recurrent anterior shoulder instability due to neuromuscular imbalance that developed as a sequel of tuberculous meningitis episode.

## 2. Case Presentation

A 12-year-old girl was admitted to our orthopaedic department for intoeing and shoulder instability. She had a previous history of tuberculous meningitis and was treated with antituberculous therapy for nine months when she was 17 months old. At that time, she was hospitalized for two months. When she was six years old, she began to feel snapping, locking, and pain in her right shoulder, which was apparent at external rotation and abduction, and her symptoms were aggravated at the last one year.

**Figure 1:**
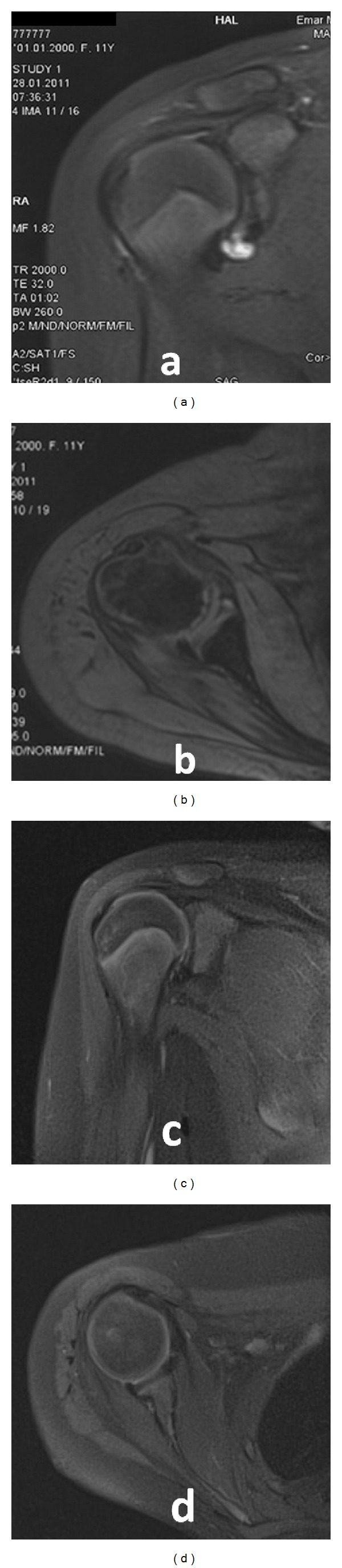
Preoperativecoronal (a) and axial (b) MRI views of capsulolabral separation. Postoperative one-year coronal (c) and axial (d) MRI views of capsulolabral repair.

She complained especially of pain in her right shoulder for overhead activities. In the physical examination of her shoulder, painful anterior inferior instability was observed with 90 degrees of abduction and 70 degrees of external rotation. Her shoulder also dislocated with 60 degrees of external rotation in a neutral position and after 120 degrees of forward flexion. Apprehension and fulcrum tests and sulcus sign were all positive. Neurovascular examination, deep tendon reflexes, and electromyography findings were normal. Shoulder radiographs and CT scan showed no bony pathology or any glenoid deficiency. A capsulolabral tear could be seen with MRI (Figures 1(a) and 1(b)).

**Figure 2:**
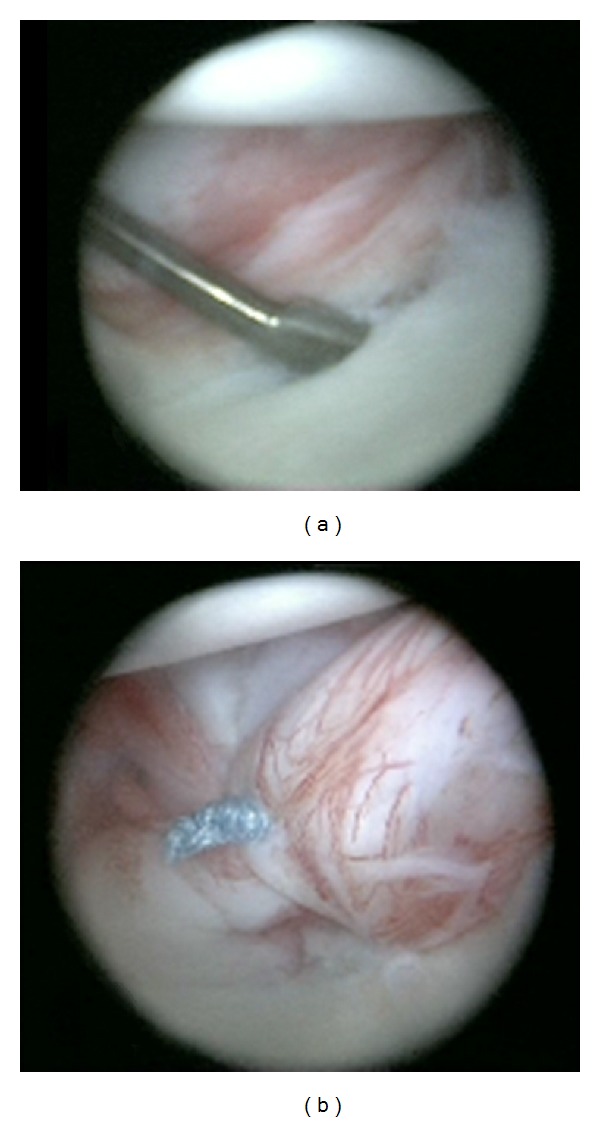
Arthroscopic views of the lesion (a) and after capsulolabral repair and rotator interval closure (b).

**Figure 3:**
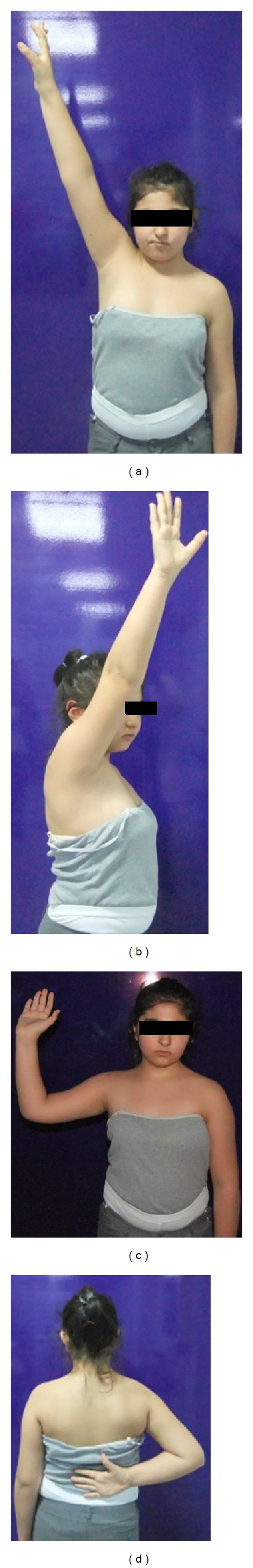
Range of motion and functional clinical views of the patient.

Detailed information on surgical interventions was provided to the patient and her family. An informed consent form concerning the operative techniques to be performed was signed by her parent. She and her family were enlightened about the rehabilitation program to be instituted. We performed shoulder arthroscopy to identify the Bankart lesion and to detect the presence of associated possible lesions through standard posterior, anteroinferior, and anterosuperior portals in the lateral decubitus position (Figure 2(a)). No signs of joint tuberculosis nor synovial problems were detected. We performed inferior capsulolabral repair with a 3 mm knot tying suture bioanchor (Bio-corkscrew, Arthrex, Naples, FL, USA). Rotator interval closure was done in order to obtain absolute stability (Figure 2(b)). We also made a dynamic shoulder stability examination with arthroscopy at the end of the surgery. On the second postoperative day, the patient was directed to the physical therapy department with a shoulder sling. Isometric shoulder-strengthening exercises were provided for six weeks. The sling was removed at week six after surgery, and passive range of motion was started. Active-assisted and active exercises were started at week eight postoperatively. The intensity and frequency of the scapulothoracic strengthening exercises were gradually increased; rotator cuff strengthening exercises were done in a side-lying position, and active, resistive strengthening and stretching exercises were begun 10 to 12 weeks postoperatively. At her one-year followup, there was no history of instability nor a dislocation recurrence. Provocative tests became negative, and forward flexion was 170°, abduction was 150°, and external and internal rotations were 70° (Figure 3). Outcomes of the surgery were evaluated with the Rowe Shoulder Instability Score [8]. The Rowe score increased from 25 points (poor) at preoperative to 90 points (excellent) at postoperative sixth month. Anterior inferior capsulolabral complex was detected to be completely intact and restored on a postoperative MRI control (Figures 1(c) and 1(d)).

## 3. Discussion

Dislocation of the shoulder joint in children is very rare. Rowe reported only eight paediatric patients in a series of 500 cases [9]. Traumatic anterior dislocations in juvenile patients were presented with good results after closed reduction and conservative treatment [10]. Congenital, paralytic, and spastic shoulder dislocations in newborns were initially treated conservatively, and surgery was performed in some cases of failure following therapy [11]. Congenital dislocations are present at birth and are associated with abnormal development of the shoulder girdle, neglected septic arthritis, or congenital abduction contracture of the deltoid muscle [1, 12].

Neuromuscular causes of shoulder instability in children have been reported as recurrent posterior dislocations, developed after brachial plexus birth palsy and cerebral palsy, and have been treated by arthroscopic and open procedures [3, 5]. Different surgical techniques for the management of neurologic posterior dislocation of the shoulder as a result of upper brachial plexus birth injury were presented, such as derotational humeral osteotomy, muscle transfers, and arthroscopic subscapularis release [13–15]. Anterior dislocation of the shoulder is rarely associated with obstetric paralysis, and all of these cases are treated conservatively and additional physiotherapy is used to prevent contracture [4].

Neuromuscular dislocation of the shoulder following central nervous system infections is exceedingly rare. Percy described a woman in whom posterior dislocation developed after an episode of encephalitis [3]. Another case of anterior subglenoid dislocation of the shoulder in a seven-month-old boy following pneumococcal meningitis was reported [7].

The glenohumeral joint, because of its poor osseous and capsuloligamentous stability, necessitates an assurance on musculotendinous proprioception and stabilization under upper central nervous system control [16]. Surgical procedures and rehabilitation protocols provide successful functional outcomes by restoring normal musculotendinous, capsuloligamentous, and neuromuscular integrity.

Our patient had recurrent anterior dislocation that developed after an episode of tuberculous meningitis in infancy. Dislocation of the shoulder was associated with neuromuscular coordination deficiency after infectious brain damage. The anterior inferior glenohumeral capsulolabral complex was repaired with rotator interval closure performed by shoulder arthroscopy. Good functional results and no recurrence of instability were achieved postoperatively after a rehabilitation protocol. This is the first case report of a recurrent anterior shoulder instability that occurred as a sequel of tuberculous meningitis in a child.

Central nervous system infections in infancy may be the reason for shoulder instability during childhood. This situation causes a disability for children and can be treated successfully with shoulder arthroscopic stabilization and effective postoperative rehabilitation.
